# *Helicobacter pylori* Oncogenicity: Mechanism, Prevention, and Risk Factors

**DOI:** 10.1155/2020/3018326

**Published:** 2020-07-12

**Authors:** Muzaheed

**Affiliations:** Department of Clinical Laboratory Science, College of Applied Medical Sciences, Imam Abdulrahman Bin Faisal University, Dammam, Saudi Arabia

## Abstract

*Helicobacter pylori* (*H*. *pylori*) is the most common cause of gastric ulcer; however, its association with gastric cancer has been proved through a variety of studies. Importantly, *H*. *pylori* infection affects around half of the world's population leading to a variety of gastric problems and is mostly present in asymptomatic form. Although about 20% of people infected with *H*. *pylori* develop preneoplastic gastric lesions in later stages of their life, around 2% of infected individuals develop gastric cancer. Nevertheless, the outcome of *H*. *pylori* infection is determined by complex interaction between the host genetics, its environment, and virulence factors of infecting strain. There are several biomarkers/traits of *H*. *pylori* that have been linked with the onset of cancer. Among these, presence of certain major virulence factors including cytotoxin-associated gene A (CagA), vacuolating cytotoxin (VacA), and outer inflammatory protein A (OipA) plays a significant role in triggering gastric cancer. These factors of *H*. *pylori* make it a potent carcinogen. Therefore, eradication of *H*. *pylori* infection has shown positive effects on decreasing the risk of gastric cancer, but this has become a challenge due to the development of antibiotic resistance in *H*. *pylori* against the antibiotics of choice. Thus, the unmet need is to develop new and effective treatments for *H*. *pylori* infection, considering the antimicrobial resistance in different regions of the world. This review discusses the properties of *H*. *pylori* associated with increased risk of gastric cancer, antibiotic resistance pattern, and the possible role of eradication of *H*. *pylori* in preventing gastric cancer.

## 1. Introduction

*Helicobacter pylori* (*H*. *pylori*) is a Gram-negative pathogenic bacterium and its infection causes inflammation of the stomach tissues leading to gastric ulcer. If not treated properly, it can result in a lifelong infection or predispose individuals to gastric cancer. Gastric cancer is one of the most common cancers all over the world [1]. It is the third most fatal type of cancer and is responsible for a large number of cancer-related deaths. However, the most worrisome aspect is that 75% of gastric cancer cases are due to *H*. *pylori* infections [2]. But the reduction lately in the *H*. *pylori* infection led to a relatively lower incidence of gastric cancer [3]. Several scientific studies have tried to decipher factors associated with the overall epidemiology, mainly focusing on the onset of gastric cancer, prognosis, and treatment outcomes. There is a need to understand why cancer has a relatively lesser prevalence but causes such a high level of morbidity and mortality. Importantly, several genetic and epigenetic factors have been linked with the overall pathogenesis of this bacterium.

Besides, appropriate control of infection with antibacterial, earlier diagnosis, and appropriate public health measures assisted in reducing the burden of gastric cancer in high-risk areas of *H*. *pylori*, particularly in developing countries. However, further research is needed to better understand the pathogenesis of *H*. *pylori* for minimizing and controlling the complications, related to this pathogen, including gastric cancer. The aim of the current review is to highlight the importance of early detection of *H. pylori* infections, which could have a great impact on protection from gastric cancer as a result of the early intervention and successful eradication of *H*. *pylori* before eliciting gastric cancer. Also, this review exposes the challenge of the emerging strains of antibiotic-resistant *H*. *pylori* to the scientific community to recommend the need to develop novel antibacterial agents.

## 2. Gastric Cancer Types and Its Prevalence

In general, gastric cancer is considered as the fifth most common cancer across the world. However, as far as the relative mortality is concerned, it is the third most fatal type of cancer worldwide and is responsible for a vast number of cancer-related deaths [1]. Gastric cancer classification is based on the types of histological features. There are two major types of gastric malignancies: adenocarcinoma and lymphoma of gastric mucosal-associated lymphoid tissue (MALT). The gastric MALT is less common than adenocarcinoma and accounts for only 3% of all gastric tumors. *H. pylori*-associated chronic gastritis is the underlying cause of both of these malignancies. Of note, most of the gastric cancer cases that were associated with *H*. *pylori* infections were MALT lymphomas [4]. It has been reported that around 70% of all cases of gastric cancer globally are caused by *H*. *pylori* infections [5].

Unfortunately, trends are quite disturbing regarding geographical stratification of varied prevalence. For example, the incidence of gastric cancer is high in some areas, including Central and South America, Eastern Europe, and East Asia. Varied geographical prevalence of the *H*. *pylori* is quite logical also as the bacteria survive in the acidic environments of the human stomach. People have different food habits varying with the regions. As such, in the days ahead, areas having a higher prevalence of gastric cancer need to be evaluated in the backdrop of dietary habits also. Gender preference has been reported through scientific studies, and men are more prone to contract gastric cancer as compared to women [6]. The underlying cause of men's predisposition to this specific cancer is an active area of investigation.

### 2.1. Major Risk Factors of Gastric Cancer

Several studies have reported on the role of different risk factors in triggering the pathogenesis of gastric cancer. Based on the type and nature, gastric cancer risk factors have been categorized as three main types.

#### 2.1.1. Genetic Risk Factors

The genetic basis of gastric cancer has been extensively studied. It has been found that there are specific genetic polymorphisms strongly linked with gastric cancer, in addition to the presence of certain gene mutations that determine the genetic predisposition to the disease. In this regard, it has been reported that a relationship exists between gastric cancer and many cytokine gene polymorphisms. Several studies allude to the fact that single nucleotide polymorphisms in interleukin genes like *IL-10T-819C*, *IL-8-251*, *IL-18RAP917997*, *IL-22 rs1179251*, *IL1-B-511*, *IL1-B-3954*, *IL4R-398*, and *IL1RN* are correlated with premalignant gastric lesion susceptibility [7]. Besides interleukins, polymorphism changes in several other biological moieties are also linked with gastric cancer predisposition. For example, the polymorphism of CYP19A1 gene, especially single nucleotide polymorphisms (SNPs) rs16964228, was found to be associated with an increased risk of gastric cancer [8]. Similarly, the genetic polymorphisms of NAT2 M1, CYPE1, and XRCC1 194 genes are also associated with gastric cancer [9].

A variety of genetic factors are faithfully transmitted from parents to their offspring. As such, the involvement of various genetic features suggests the familial disposition of gastric cancer based on the type of genetic architecture they inherit from their parents. Therefore, the family history of the disease has been considered as a significant risk factor of gastric cancer. The first evidence of the relationship between family history and inheritance of gastric cancer was reported in the year 2012—high prevalence of gastric cancer in people whose first-grade relatives have suffered from the same disease [10].

A comparative study of genetic similarity among affected individuals has provided valuable information about the molecular genetic pathways that are linked to the increased risk of gastric cancer in families with a history of this malignancy [11]. Previously, it has been reported that the risk of abdominal cancer in people with a family history of this disease was around ten times higher than that of people with no family history [12]. Considering the transmission of gastric cancer susceptibility through hereditary mechanisms, germline genetic testing has been recommended as far as the issue of gastric cancer preventive measures is involved [13].

#### 2.1.2. Environmental Risk Factors

Among environmental factors linked with gastric cancer, exposure to radiation, toxins, cigarette smoke, and lifestyle are the most important environmental risk factors. Smoking is associated with many kinds of human cancers, including lungs, blood, and mouth cancers [14]. It has also been found to be strongly linked to gastric cancer, as proven by several researches [15, 16]. Several meta-analyses and observational studies allude to the fact that smoking predisposes individuals to gastric cancer [17]. New data also corroborates with previous findings. For example, a Japanese study concludes that past and current smokers tend to have an increased risk of a specific type of gastric cancer, which is in the distal region, as compared to nonsmokers. The relative risk of gastric cancer in smokers was found to be higher as compared to nonsmokers, respectively [15].

There was a gender difference in gastric cancer incidence with smoking, and it was observed that men are more susceptible compared to smoking women [18]. Lifestyle includes the attitudes, interests, characteristics, and behavior of an individual or culture and refers to the lifestyle of a person or community [19]. Diet, physical activity, personal hygiene, and education are vital parts of lifestyle, and various studies have supported the role of lifestyle as a risk factor of gastric cancer [20].

#### 2.1.3. Biological Risk Factors

The list of biological risk factors associated with human cancer incidence is dynamic. A variety of pathogens have been identified as potential cancer-causing agents in humans. For instance, viral infections causing cancer in human account for 10–20% of cancers worldwide. Several epidemiological and experimental studies confirmed the relationship between the human papillomavirus and anal cancer. Likewise, Epstein–Barr virus and John Cunningham Virus (JCV) are likely causes of cancer in upper and lower gastrointestinal tract [21]. Similarly, parasitic diseases like schistosomiasis, opisthorchiasis, and clonorchiasis are associated with human carcinogenicity [22].

Several bacteria, such as *Streptococcus bovis*, *Salmonella typhi*, and *Chlamydia pneumoniae* have associations with different types of neoplasms, although their possible role in carcinogenesis is unclear [23]. A recent case-control study reported that *H*. *pylori*, *Propionibacterium acnes*, and *Prevotella copri* are strong risk factors for development of gastric cancer; however *Lactococcus lactis* is a protective factor [24]. Amongst biological risk factors, the presence of *H*. *pylori* infection is the most significant risk factor of gastric cancer, and many studies have investigated the aspects of the relationship between *H*. *pylori* infection and gastric cancer [25, 26].

## 3. *Helicobacter pylori*

*H.pylori* is a most common pathogen of the human gastric tract and causative agent for various pathologies, including chronic gastritis and peptic ulcers, and contributes to gastric cancer development. *Helicobacter* species are subdivided into two major groups: gastric and enteric. Indeed, gastric *Helicobacter* species is considered as the most successful human pathogen, which is *H*. *pylori*. The infection is mostly acquired during childhood period and remains for decades and may be a lifelong infection. The severity of the clinical outcome of *H*. *pylori* infection is mainly influenced by the complex interaction between the host, its environment, and bacterial factors in a specific population [27].

### 3.1. *H*. *pylori* and Gastric Cancer

The outcome of *H*. *pylori* infection can be atrophic gastritis that can cause gastric carcinoma, or it can remain localized and limited to duodenal ulcer disease. The most significant factor which governs this outcome is controlled by the polymorphisms or mutations present in the genes of the host. These mutations regulate the intensity of the inflammation in the gastric tissue that impacts the risk of specific clinical effects and outcomes [28].

Several studies have shown a nexus between *H*. *pylori* and gastric lymphoma; however, the most intriguing one is by an earlier intriguing study. According to the findings of this study among patients suffering from this type of stomach cancer, CagA protein is the deciding factor. Histologically, stomach lymphomas can be either low-grade lymphoma of MALT type (LGLM) or diffuse large B-cell lymphoma (DLBCL). It was observed that, among the 53 patients evaluated in the study being discussed here, 45 were *H*. *pylori*-positive, thus reflecting an association of the bacteria with this type of cancer. Importantly, the majority of the patients having DLBCL were *cagA*-positive [29].

It has also been reported that the clinical symptoms and presentation of the infection of *H*. *pylori* determine the pattern and seriousness of gastritis. An example is individuals who are *H. pylori*-infected and live in areas where seasonal diets are typical dietary ingredients. In these areas, there is a long period without the availability of fresh vegetables and fruits along with food preservation by using salt and smoking, which are found to be at a higher risk of developing progressive gastric atrophy. Progressive atrophy of stomach is associated with gastric ulcers and gastric cancer, and those individuals are more prone to develop gastric cancer due to *H*. *pylori* infection at some stages of their life [30]. In contrast to these findings, the availability of fresh fruits and vegetables in some regions of the world, including South Asia, Africa, and South India, has revealed relatively lower incidence of gastric cancer as a result of *H*. *pylori* infections, which mostly lead to duodenal ulcer and related complications instead [31]. A variety of other factors define the risks of gastric cancer in *H*. *pylori* patients. These factors are widely studied and are discussed in the following.

### 3.2. Family History

There exists a strong relationship between gastric cancer and the family history of interaction with *H*. *pylori* infection leading to the progression of gastric cancer. A definitive interaction occurs at the genomic and physiological level between the genetic risk factors of gastric cancer and *H*. *pylori* pathogenesis; this is relatively higher among individuals with a positive family history of the infection as compared to those without the exposure. Persons with a family history of *H*. *pylori* infection are at a 5-fold increased risk of developing gastric carcinoma at some stage in their lives [32]. Along with similar lines, a recent study manifests that having one of the parents who has gastric cancer enhances the chances of acquiring this type of cancer significantly [33].

### 3.3. Virulence Factors

There are many traits and features of the *H*. *pylori* that make it a potent carcinogen. First, it invades and infects the body like a regular pathogen and causes inflammation in its target tissues. However, there are specific mechanisms of pathogenesis that may lead to serious health consequences, including gastric cancer. Important pathogenic factors include its potent virulence factors and cell-damaging molecular mechanisms. Due to its pathogeneses, gastric cancer is recognized as a model for inflammation-induced cancer [34]. Several studies have been conducted to understand the mode of action of *H*. *pylori* on gastric tissues and their cancer-causing effects. Virulence factors are considered as additional pathogenic factors of an invading microbe that increase the cellular damage of the host. Several reports have investigated the virulence factors of *H*. *pylori* and their potential role in triggering gastric cancer. According to Yamaoka and Graham [26], the most important virulence factors of *H*. *pylori* are CagA, VacA, and OipA, besides several others, as outlined in Table 1. The CagA is a potent immunogenic protein. *H*. *pylori* with *cagL* gene have better adhesion abilities, where *cagL* promotes the transfer of CagA protein into the target cells. Inside the host cell, the CagA protein interacts with the cytoplasmic SHP-2, which is an oncogenic protein [26]. This protein (SHP-2) is also known to increase the intensity of inflammation in the gastric tissues—a key risk factor for triggering gastric cancer [46].

The VacA has also been linked to the increased pathogenesis of *H*. *pylori* reflected in the severity of clinical outcomes. Mainly the VacA protein induces the production of endosomal vacuoles in gastric epithelial cells, promotes apoptosis, and inhibits T-cell proliferation in vitro. These features are associated with significant gastric mucosal damage and immune dysregulation, both of which can lead to gastric carcinoma [47]. An Italian study on the prevalence of VacA protein has shown that another variable region within the vacA gene, termed the i region, has been associated with increased gastric cancer risk [48]. Similarly, OipA is an adhesion protein present on the outer membrane of the *H*. *pylori*. It has been reported that this adhesion protein plays an essential role in the virulence of *H*. *pylori* and determines the inflammatory response. In addition, OpiA interacts with various cell-signaling pathways of the host [49]. Most of these signaling pathways regulate cell-cell junction and cellular proliferation; therefore, dysregulation of these pathways by OpiA can negatively affect the homeostasis of cell division and growth, which in turn increases the risk of gastric carcinomas [50].

## 4. Mechanisms of *H*. *pylori* That Lead to Gastric Cancer

*H. pylori* colonizes the human stomach, thus promoting the pathogenesis of bacteria in the gastric system. During the course of bacterial pathogenesis, there is further development of chronic atrophic gastritis, intestinal metaplasia, and gastric cancer. The *H*. *pylori* causes inflammation of the stomach tissues, and if not treated properly, it can result in a lifelong or chronic infection. Various mechanisms favor the survival of *H*. *pylori* in the pylorus gastric environment. For instance, it produces a urease enzyme that aids in the buffering of acidic pH of the stomach by the production of ammonia from urea [51]. *H. pylori* has a distinct flagellum that helps in its movement, and its helical shape facilitates its invasion in the mucous layer of gastric epithelial cells [52].

It has been reported that early stages of gastritis and atrophy have been linked with consumption of excessive salt and infection with *H*. *pylori*. The intermediate stages have been linked with ascorbic acid and nitrate consumption that aggravate the damaging effects of inflammatory response elicited by *H*. *pylori*. The final stages of *H*. *pylori* pathogenesis have been linked with the level of *β*-carotene and increased consumption of salty food [53].

### 4.1. Precancerous Conditions

A precancerous lesion of the stomach is an abnormal histopathological condition possibly leading to gastric cancer. The precancerous conditions have been linked with long-term mucosal infections with *H*. *pylori*. This infection comprises various phases and with passage of time each stage involves a marked progression in damage to the gastrointestinal tract. These precancerous conditions have been divided into five stages: active nonatrophic gastritis (chronic gastritis caused by inflammatory response against *H*. *pylori*), multifocal atrophic gastritis (permanent damage to epithelial cells and gastric gland due to prolonged inflammation), intestinal metaplasia (complete/incomplete), dysplasia (cytological abnormalities of the epithelium), and finally invasive carcinoma [54, 55]. In this context, the progression of different stages and the appearance of precancerous lesions are modulated by various factors including diet and high intake of salt that increase the risk; meanwhile high intake of fruits and vegetables lowers the risk.

### 4.2. Alteration of the Balance Proliferation/Apoptosis of Gastric Epithelium

A very intricate molecular mechanism maintains the integrity of gastric mucosal cells. This integrity is regulated by certain equilibrium between cell proliferation and loss of cells due to programmed cell death—apoptosis [56]. Under normal conditions, apoptotic cells are rare in stomach; but infection with *H*. *pylori* increases the rate of apoptosis, especially in the gastric gland. Moreover, the presence of *H*. *pylori cag* pathogenicity island, which expresses cagA, adds to this alteration of balance. CagA increases the proliferation of gastric cell and *cagA*-positive strains reduce apoptosis. This imbalance in the gastric cell number leads to an increased risk of gastric carcinoma in individuals who are infected with *cagA*-positive *H*. *pylori* strains [57].

### 4.3. Molecular and Biochemical Mechanism

**Figure 1:**
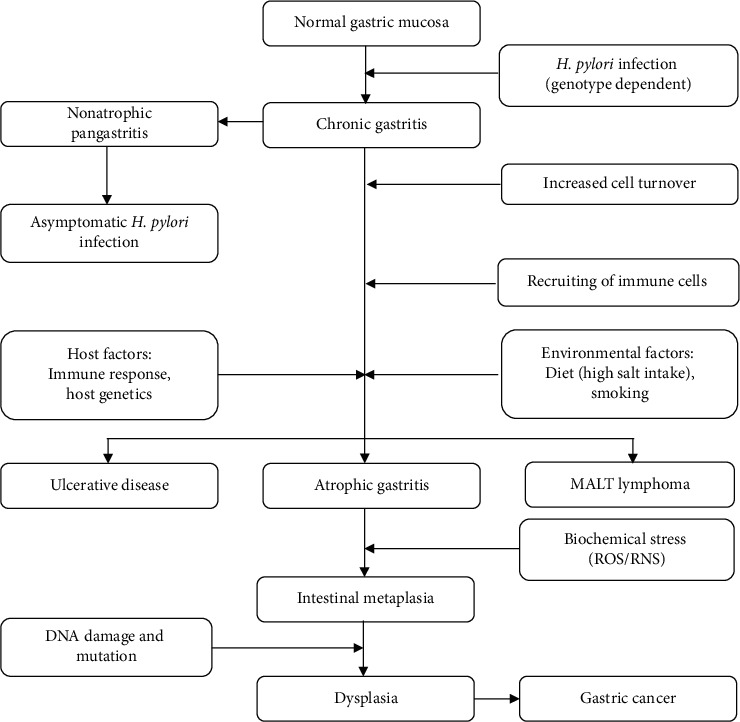
Schematic representation of the causative factors of gastric cancer and diseases induced by *H*. *pylori* infection.

There are specific cellular mechanisms that are induced by the infection and the concomitant inflammation of gastric cells as a result of *H*. *pylori* infection. It has been reported that two of the most common biochemical mechanisms that play a significant role in *H*. *pylori*-induced gastric carcinogenesis are the induction of oxidative and nitrosative stress in the host cells [58]. This happens through a variety of molecular changes as a consequence. The pathophysiology of *H*. *pylori* infection and other factors contributing to causing gastric cancer are represented in Figure 1.

These mechanisms cause serious cellular damage and DNA insult. The presence of continuous stress on the cell leads to the depletion of antioxidant defenses and damage-repair mechanisms. This causes a surge in the genetic errors (mutations) that arise under the pressure of increased turnover or division of gastric epithelial cells [59]. These mutations accumulate in the gastric cells overgeneration and lead to the formation of malignant tumors that are manifested as gastric cancer. In this way, *H*. *pylori* infection causes oxidative damage to cells, which in turn increases the risk of cancer [60]. The intrinsic reactive oxygen species (ROS) production is influenced by the virulence factors from *H*. *pylori*, including VacA and CagA, which are introduced into the epithelial cells of the stomach [58].

The intrinsic ROS production is responsible for activating apurinic/apyrimidinic endonuclease 1 (APE1), which then translocate to the nucleus to mediate the repair of damaged DNA. Spermine oxidase (SMO) is also stimulated and leads to DNA damage. Recruiting immune cells to the area as driven by the virulence factors, such as NapA, produces additional extrinsic ROS in an effort to clean up the infection, but this further damages the cell and increases the inflammation in the gastric tissue. The prolonged oxidative stress, increased inflammation, and the failure of the immune system to remove *H*. *pylori* from the gastric tissues lead to inevitable tumorigenesis [58]. Also, it has been reported that endoplasmic reticulum (ER) stress and the unfolded protein response (UPR) are linked to the pathogenesis of *H. pylori* leading to gastric tumorigenesis.

### 4.4. Inflammatory Response

In general, inflammatory response helps in controlling infectious organisms; however, it is quite different as far as the *H*. *pylori* is concerned as it paves the way for bacterial colonization to induce gastric mucosal oncogenesis process. Scientific studies have shown that gastric cancer mostly develops in patients who have the most robust inflammatory response to *H*. *pylori*. Bacterial colonization induces vigorous humoral and cellular responses through the recruitment of immunoinflammatory cells, including lymphocytes, neutrophils, plasma cells, and eosinophils, followed by damage to epithelial cells of the stomach. The bacterial virulence factors elicit those responses in host gastric epithelial cells that are present in the gastric pits, which lead to carcinogenesis. A robust immune response leads to the production of proinflammatory cytokines. These cytokines have both beneficial and detrimental effects on the host [61]. Two models depict the role of cellular inflammation in *H*. *pylori*-induced cancer:

  Inflammation causes increased turnover of epithelial cells, thus increasing the chance of mutations, ultimately reflected in cancer development [62].
 
*H*. *pylori* species act as a trigger of a chronic inflammatory response that leads to the recruitment of bone marrow-derived cells to the gastric epithelium. These cells directly contribute to malignancy in the gastric tissues [63].

The adaptive biochemical mechanisms are triggered as a result of *H*. *pylori* infection, including oxidative stress, ER stress, autophagy, and inflammation. This response leads to the formation of a precancerous lesion in the gastric tissues [64]. ER stress induced by *H*. *pylori* infection is the primary cause of early stages of precancerous lesions formed in the gastric tissues [65]. It has been reported that the ER stress sensor, protein kinase RNA-like endoplasmic reticulum kinase (PERK), plays an essential role in the progression of the tumor by increasing the invasion and migration rate of gastric cells. Adding to that, the abnormal increase in the growth of epithelial cells leads to the formation of a solid tumor mass [66]. In contrast, the prolonged and chronic *H*. *pylori* infection represents a continuous stimulus of ER stress-mediated carcinogenesis that results in apoptosis [67].

Additionally, *H*. *pylori* takes advantage of NF-*κ*B activation that leads to the development of a long-term, chronic infection. The accumulation of autophagosomes in the epithelial cells that are triggered by the precancerous cascade exaggerates oxidative stress (via the production of ROS) that supports cancer aggressiveness and metastasis [68]. Besides this, long-term inflammation of the gastric tissue generates large amounts of nitric oxide, which is also damaging to cells. In this way, the inflammatory response elicited by *H*. *pylori* favors cancer progression if the infection is not treated.

### 4.5. Animal Models of *H*. *pylori* Pathogenesis

Various animal models have been developed to mimic and investigate the pathogenesis of *H*. *pylori* since the discovery of this pathogen. The inoculation of *H*. *pylori* was reported to induce gastritis in mice, miniature pigs, beagle dogs, Japanese monkeys, and Mongolian gerbils. These studies showed that *H*. *pylori* infection has the potential to induce histologic gastritis [69, 70].

One of the earliest studies on animal models developed to examine the association between *H*. *pylori* infection and gastric cancer was by Hirayama in 1996 [71]. They reported that, in Mongolian gerbils, prolonged *H*. *pylori* infection can lead to induction of gastritis, gastric ulceration, and intestinal metaplasia. The timeline of infection was also analyzed, and it was found that *H*. *pylori* could colonize the stomach and induce gastritis in 3 months after first inoculation. In addition, gastric ulceration occurs within 6 months and intestinal metaplasia after 6 to 12 months. Several recent studies on animal models have highlighted the role of *H*. *pylori* in inducing gastric inflammation in gerbils [72, 73]. Long-term infection of *H*. *pylori* not only caused precancerous lesions in gerbil gastric mucosa, but also resulted in gastric adenocarcinoma [74]. The role of cag pathogenicity island in the development of *H. pylori*-induced disease in various animal models, including mice and rhesus monkeys, supports the significant effects of cytotoxin-associated gene A (CagA) and other related factors in causing gastric cancer [75].

### 4.6. Prevention of Gastric Cancer by Controlling *H. pylori*

Several studies have been conducted in the past to assess whether and to what extent the damage of the inflammatory process of *H*. pylori, including atrophic gastritis and intestinal metaplasia, can be reversed by eradication of bacteria. The endoscopic analysis of *H. pylori*-positive gastric samples manifests the presence of enlarged or elongated pit patterns; an advance in the texture of gastric tissue with the appearance of an oval, small, or round pits was observed along with decreased densities of irregular vessels [76]. In this context, atrophic gastritis occurs in the early stages of *H*. *pylori* infection, which can lead to irreversible intestinal metaplasia [77].

It is now established that the gastric cancer risk depends on the type and the severity of baseline precancerous lesion. A study from the Netherlands has reported results from 5 years' follow-up of patients who suffer from low- or high-grade dysplasia and showed that there was a 0.6–6.0% risk of cancer in these patients per year, respectively. On the other hand, patients who develop atrophic gastritis and intestinal metaplasia as a result of *H*. *pylori* infection had 0.1% and 0.25% annual risk to develop stomach cancer, respectively [78].

A randomized, controlled study carried out in Japan revealed that *H*. *pylori* eradication causes a reduction in the incidence of gastric cancer during a 3-year follow-up period [79]. Another study showed that the occurrence of gastric cancer after endoscopic resection of early gastric cancer is significantly lower in the *H*. *pylori*-free group as compared with the control noneradicated group [80]. Similarly, *H*. *pylori* eradication therapy decreased the risk of primary gastric cancer in healthy asymptomatic infected individuals when compared with the corresponding control individuals [81].

Various studies have reported the positive effects of *H*. *pylori* eradication in effectively improving the gastric lesions in the corpus or antrum and reducing the symptoms of gastritis [28, 82]. Eradication of *H*. *pylori* is associated with the decreased risk of gastric cancer, where the endoscopic analysis of stomach cancer samples has shown that the gastric tumor regions have a gastritis-like appearance instead of having typical malignant characteristics [83]. Furthermore, histopathological analysis of gastric dysplasia showed substantial alterations in the morphology of the tumor and its characteristics after the eradication of *H*. *pylori* [84].

All these studies have shown that eradication of *H*. *pylori* is very significant for the protection from gastric cancer. We can infer that early eradication of *H*. *pylori* is the best approach, which can help to prevent gastric cancer. In contrast, a few studies have reported the probability of incidence and development of stomach cancer at later stages in life in patients who have undergone *H*. *pylori* eradication therapy [85]. Some studies reported that around 1.0% of patients had gastric cancer even though they were successfully eradicated of *H*. *pylori* infection [66, 86]. It has also been reported that gastric tumors could develop even after several years of *H*. *pylori* eradication [76, 87]. One possible explanation for this finding is that gastric cancer and other tumors are a multifactorial disease. The elimination of one factor does not guarantee prevention in all causes of gastric cancer [88]. Generally, it is advised that people should get regular endoscopic surveillance, especially patients who suffer from severe gastritis and are at high risk due to the involvement of other genetic and environmental factors [89].

### 4.7. Antibiotic Resistance in *H*. *pylori*

The treatment of *H*. *pylori* is challenging itself; however, the development of antibiotic resistance further exacerbates the situation. Several studies were conducted to determine the prevalence, mechanism of resistance, different methods of detection, and the clinical consequences of resistance. Most common antibiotic-resistant strains of *H*. *pylori* are clarithromycin- and metronidazole-resistant, and they are present at very high rates but vary among different populations. However, tetracycline and amoxicillin resistance are relatively lower. The percentage resistance of *H*. *pylori* against various antibiotics widely varies from different geographical regions. North Americans have the highest resistance against clarithromycin above 30 percent, whereas, in the African continent, relatively higher resistance has been observed against amoxicillin, tetracycline, and metronidazole: 41, 50, and 75 percent, respectively. In contrast, the Asian population manifests higher resistance levels to levofloxacin [90]. These trends are quite intriguing and serve as benchmark as far as the treatment of *H*. *pylori* infection is concerned.

Various genetic determinants are involved in imparting antibiotic resistance in *H*. *pylori*. Genome-wide association studies (GWAS) revealed several genes responsible for developing resistance in *H*. *pylori* against various antibiotics. A recent study conducted on 140 clinical *H*. *pylori* isolates that compared phenotypic antibiotic susceptibility testing (AST) results with the presence of predicted genetic determinants using whole genome sequencing (WGS) found more than 99% similarity between phenotypic antibiotic susceptibility testing for clarithromycin, levofloxacin, and rifampicin and certain SNPs identified in the 23S rRNA, *gyrA*, and *rpoB* gene [91].

Various molecular and phenotypic methods have been developed to detect *H*. *pylori* resistance. The use of genomic techniques has opened new possibilities in the diagnosis of *H*. *pylori*. Also, the use of stool samples for the detection of *H*. *pylori* and its antimicrobial resistance is a feasible and noninvasive approach. In this regard, the rate of eradication is dependent on the susceptibility of the strain to metronidazole and clarithromycin, and it is low in patients who are infected with a resistant strain of *H*. *pylori* [92]. The World Health Organization (WHO) declared clarithromycin-resistant *H*. *pylori* strain as “high priority” for which new antibiotics need to be explored although different regions have differential and several resistance developments against conventionally used antibiotics for the management of *H*. *pylori*. Additionally, susceptibility tests should be a prerequisite for treating patients suffering from *H*. *pylori* issues, mainly for the areas having its higher incidence.

## 5. Conclusion

*H.pylori* infections in humans have been a challenge due to a higher incidence rate of the disease and the development of antibiotic resistance. Failure to eliminate *H*. *pylori* infection is associated with severe health consequences, including gastric cancer. Mainly, this specific type of cancer has very high morbidity and mortality. Conclusive studies highlight the crucial role of *H*. *pylori* infection in triggering gastric cancer. Various virulence factors and metabolic processes of *H*. *pylori* play a very significant role in developing gastric carcinogenesis.

Studies should be focused on developing inhibitory mechanisms for the *H*. *pylori* infection and proper measures should be taken to eradicate this bacterium from infected individual along with usage of appropriate antibiotics. It is critical to determine the time frame in which *H*. *pylori* causes a carcinogenic effect, as after this time point, the intervention is relatively less effective. Population-wide screening program should be applied in high-risk areas for earlier identification of the asymptomatic patients and to reduce morbidity, mortality, and healthcare costs. Mainly, harnessing artificial intelligence by employing a machine learning algorithm is another avenue to be explored in the earlier diagnosis of this cancer.

## Figures and Tables

**Table 1:** Major virulence factors of *H*. *pylori* and their biochemical effects in pathogenesis.

Virulence factor	Potential biochemical effect	References
*Colonizing factors*		
Urease	Neutralizes stomach acid and urease-medicated activation of neutrophils and platelets causes gastric inflammation	[35]
Flagella	Enables the bacterium to move toward gastric epithelium cells and leads to colonization and persistent infection	[36]
Chemotaxis mechanism	Enables biofilm formation to induce oncogenic process and development of antibiotic resistance	[37]

*Cell-surface proteins (adhesins)*		
BabA	Mediates attachment to the gastric epithelial cells and induces DNA double-strand breaks	[38]
SabA	Mediates bacterial attachment and colonization	[39]
OipA	Damages gastric mucosal membrane and causes cellular apoptosis	[40, 41]

*Pathogenicity factors*		
CagA	Enhances cellular proliferation and IL-8 expression	[42, 43]
VacA	Induces cytoplasmic vacuole formation and causes cellular apoptosis	[44]
HtrA	Helps in delivery of CagA	[45]
